# The clinical impact of valvular heart disease in a population-based cohort of subjects aged 80 and older

**DOI:** 10.1186/s12872-016-0184-8

**Published:** 2016-01-12

**Authors:** Nawel Rezzoug, Bert Vaes, Christophe de Meester, Jan Degryse, Gijs Van Pottelbergh, Catharina Mathei, Wim Adriaensen, Agnes Pasquet, Jean-Louis Vanoverschelde

**Affiliations:** Division of Cardiology, Cliniques Universitaires Saint-Luc, Brussels, Belgium; Pole of Cardiovascular Research, Institut de Recherche Expérimentale et Clinique, Université Catholique de Louvain, Brussels, Belgium; Institute of Health and Society, Université Catholique de Louvain (UCL), Brussels, Belgium; Department of Public Health and Primary Care, KU Leuven (KUL), Leuven, Belgium

**Keywords:** Aged, 80 and over, Valvular heart disease, Mortality, Multimorbidity

## Abstract

**Background:**

In our ageing society, valvular heart diseases (VHD) have become an increasing public health problem. However, the lack of studies describing the impact of these diseases on the outcome of very old subjects makes it difficult to appreciate their real clinical burden.

**Methods:**

Prospective, observational, population-based cohort study in Belgium. Five hundred fifty six subjects aged 80 years and older were followed up for 5.1 ± 0.25 years for mortality and 3.0 ± 0.25 years for hospitalization. Echocardiograms were performed at baseline. The Cumulative Illness Rating Scale (CIRS) was calculated for each subject.

**Results:**

The prevalence of moderate-to-severe VHD was 17 % (*n* = 97). Mitral stenosis was more prevalent in women and an age-dependent increase of the prevalence of severe aortic stenosis was seen. The overall disease burden was higher in participants with VHD (median CIRS 3 [IQR 3–5] vs 4 [IQR 3–6] (*P* = 0.008)). Moderate-to-severe VHD, and more specifically mitral stenosis and aortic stenosis, was found to be an independent predictor of both all-cause (HR 1.42 (95 % CI 1.04–1.95)) and cardiovascular mortality (HR 2.13 (95 % CI 1.38–3.29)). Moderate-to-severe VHD was also found to be an independent predictor of the need for a first unplanned hospitalization (HR 1.43 (95 % CI 1.06–1.94)).

**Conclusions:**

A high prevalence of moderate-to-severe VHD was found in the very old. Moderate-to-severe VHD was identified as an independent risk factor for all-cause and cardiovascular mortality and as well for unplanned hospitalizations, independent of other structural cardiac abnormalities, ventricular function and major co-morbidities.

## Background

In the next several decades, the proportion of very old people living in industrialized countries will dramatically increase [1]. People aged 80 and older are indeed the fastest growing age segment in the Western World, and their numbers will peak by 2050 [2]. This forthcoming “grey epidemic” will lead to an explosion of chronic diseases and generate numerous complicated cases with multiple comorbid conditions.

In this ageing society, the burden of cardiac diseases is not only rising, but its very nature is also changing. Several population-based studies have indeed demonstrated that the prevalence of systolic dysfunction in the oldest old is steadily decreasing, whereas that of diastolic dysfunction and valvular heart diseases (VHDs) is increasing [3–6]. Survival effects and age-related changes in cardiac structure and function most likely explain this reverse epidemiology [6, 7]. Despite these observations, data on the clinical burden of VHDs in elderly subjects are scarce and mostly based on in-hospital series, which introduces an important selection bias. Although some observational studies have suggested that VHDs, and particularly aortic stenosis, negatively affect the physical capacity and mental performance of elderly patients [6], very few of these studies focused on their impact on patient’s outcome and quality of life. In the absence of relevant outcome data and evidence-based guidelines, the management of elderly patients with VHDs remains empirical and most often challenging [8]. Consequently, very old patients presenting with VHDs frequently refuse or are denied valve surgery, as the risk of cardiac surgery is often perceived to outweigh any possible clinical benefits [9–11]. Although with the advent of percutaneous treatments, these patients can nowadays be treated at a much a lower risk [8], the lack of studies describing the impact of VHDs on the outcome of very old subjects makes it difficult to appreciate the real benefits of these emerging treatments. In this context, the aim of the present study was to assess the prevalence, distribution patterns, and outcome consequences of significant (moderate-to-severe) left-sided VHDs in people aged 80 and older. Based on previous observations, we hypothesized that VHDs were not only highly prevalent but also beard significant and independent prognostic implications in very old subjects from the community.

## Methods

### Study population

The BELFRAIL study is a prospective, observational, population-based cohort study of subjects aged 80 years and older in three well-circumscribed areas of Belgium (Flanders, Brussels and Wallonia). All participants in the study gave informed consent, and the Biomedical Ethics Committee of the Université catholique de Louvain (UCL) in Brussels approved the study. The study design, methods and characteristics of the cohort were previously described in detail [12]. Briefly, 29 general practitioner (GP) centers were asked to include patients aged 80 and older. No other inclusion criteria were specified, and only three exclusion criteria were used: known severe dementia (mini-mental state examination < 15), palliative care, and medical emergency, such as acute onset heart failure or decompensated heart failure. The BELFRAIL cohort is representative in gender and age of the elderly living in Belgium [12].

### Clinical evaluation

At study entrance, the GPs used a structured questionnaire to capture information on the patients’ medical history, cardiovascular risk factors, symptoms [13], and other current medical problems. These data were subsequently used to calculate the cumulative illness rating scale (CIRS) [14, 15], which was used as a multimorbidity index. The GPs were also asked to list the drugs the patients were taking on a regular basis or as needed. Drugs were classified according to the Anatomical, Therapeutic and Chemical (ATC) classification system (at level 5, which relates to the chemical substance). Data on relevant cardiovascular medications including diuretics, potassium-sparing agents, angiotensin-converting enzyme inhibitors, angiotensin II receptor blockers, β-blockers and digitalis were also collected.

The subjects’ performance in basic activities of daily living (ADL) was assessed [12]. Cognitive functioning was measured by the Mini-Mental State Examination (MMSE). Depressive symptoms were measured in those with MMSE > 18 points by use of the 15-item Geriatric Depression Scale (GDS-15) [12].

### Echocardiography

Echocardiographic data were obtained at the subject’s home using a commercially available portable system (CX50, Philips, Andover, Massachusetts, USA). All patients underwent a comprehensive examination, including M-mode and 2-dimensional echocardiography, as well as Doppler examinations, according to the recommendations of the American Society of Echocardiography and the European Association of Echocardiography (EAE) [16]. All tests were conducted by a certified cardiologist with level 3 competence in echocardiography.

The echocardiographic images were blindly analyzed by 1 or 2 observers with at least 2 years of experience in reading echocardiographic studies. All measurements were performed off-line using the XCelera software (Philips, Andover, Massachusetts, USA). In patients with poor quality images, all measurements were performed by the 2 observers and the averaged values were reported. In patients with good image quality, data were analyzed by only one single observer. To ensure adequate reproducibility, inter- and intra-observer variability was evaluated on a random sample of good quality echocardiograms. Intraclass correlation coefficients were always > 0.90.

LV volumes and LVEF were calculated by use of the biplane Simpson method, whereas LV mass was calculated according to Devereux et al [16]. Left atrial (LA) volume was measured using the biplane area-length formula. Systolic pulmonary artery pressure was considered to be equal to the systolic transtricuspid pressure gradient as calculated by use of the modified Bernoulli equation. No attempt was made to estimate right atrial pressure.

The function of the mitral, aortic and tricuspid valves was evaluated by use of standard continuous-wave, pulsed-wave and color Doppler echocardiograph, as recommended by the EAE [17–19]. In patients with aortic stenosis, stenosis severity was quantitated by use of the peak transaortic jet velocity, the mean transaortic gradient and the aortic valve area (continuity equation). In patients with mitral stenosis, stenosis severity was quantitated by use of the mean transmitral pressure gradient and the mitral valve area (direct planimetry or pressure half-time). The severity of aortic and mitral regurgitations was assessed semi-quantitatively on a scale of 1+ to 4+ by an integrated approach that included the size of the regurgitant jet in the receiving chamber, the proximal regurgitant jet width, the size of the proximal convergence zone and, when available, the regurgitant volume and the effective regurgitant orifice area. Mild, moderate and severe degrees of valve dysfunctions were graded as recommended by the EAE guidelines [18, 19].

### Outcome measures

Two detailed follow-up questionnaires were filled in by the participating GPs after 1.4 ± 0.3 years and after 3.0 ± 0.3 years. The questionnaires included questions on mortality, cause of death and unplanned hospitalization. Mortality and cause of death were also recorded by the GPs 5.1 ± 0.3 years after baseline. The causes of death were divided into cardiovascular and non-cardiovascular causes, according to the GPs’ assessment and the subsequent review by 2 independent researchers blinded to all clinical data (BV and JD). The current study used all-cause mortality, cardiovascular mortality and first, all-cause, unplanned hospitalizations for > 24 h as outcome measures.

### Data analysis

The data analysis was performed using SPSS 20.0 for Windows (SPSS Inc., Chicago, IL, USA). Continuous variables are reported as mean ± one standard deviation (SD) or median and inter-quartile range (IQR). Categorical variables are reported as numbers and frequencies. Comparisons between different categories of subjects were performed using a one-way ANOVA, the Mann-Whitney *U* test (nonparametric data) for unpaired data or the Jonckheere-Terpstra test (trend test), where appropriate.

Survival curves were computed using the Kaplan-Meier method and compared using the log-rank Chi square test. Determination of the factors independently associated with outcome was performed by use of a Cox's proportional-hazards survival analysis. For this purpose, all clinical, angiographic, and echocardiographic variables with *p* < 0.10 in the univariable analysis were proposed for inclusion into a multivariable Cox’s proportional-hazards survival model. In order to avoid multicollinearity, the correlation coefficients between all covariates were calculated. In case of multicollinearity (r-value >0.80), only one of the two covariables was considered in the multivariable model. Variable selection was performed using a stepwise, forward conditional selection procedure using the maximum partial likelihood ratio chi-square statistic (χ^2^ test) to enter (<0.05 level) or to remove (>0.05 level) a covariable into the model. Variables were entered until no F-to-enter statistics were significant at the 5 % level and until the mean squared error reached a minimum.

## Results

### Baseline clinical, hemodynamic and echocardiographic characteristics

Echocardiograms were obtained in 556 subjects (98 %), aged 84.7 ± 3.6 years, of whom 41 % were older than 85 and 9 % older than 90 years. The prevalence of moderate-to-severe VHD was found to be 17 %, and was similar in men (16 %) and women (18 %). Higher rates of MS were noted in women than in men (4.6 vs 1.5 %, *p* = 0.026). The prevalence of the other VHD was quite similar between men and women (13 vs 15 % for AS, 1.5 vs 1.7 % vs AR and 0.5 vs 0.9 % for MR). A significant trend in age-related prevalence was found for aortic stenosis, with a significantly increased prevalence of severe stenosis and a significantly decreased prevalence of mild stenosis in the very old (Table 1).

**Table 1:** Prevalence of valvular heart disease

	80–84 years	85–89 years	≥90 years	p for trend^a^
Participants, n	323	183	50	
Male, n (%)	125 (39 %)	68 (37 %)	13 (27 %)	
Mitral stenosis, n (%)	10 (53 %)	6 (3 %)	3 (6 %)	
Mild, n (%)	5 (2 %)	3 (2 %)	1 (2 %)	0.85
Moderate, n (%)	4 (1 %)	3 (2 %)	2 (4 %)	0.28
Severe, n (%)	1 (0.3 %)	0 (0 %)	0 (0 %)	0.41
Mitral regurgitation, n (%)	236 (73 %)	140 (77 %)	39 (78 %)	
Mild, n (%)	234 (72 %)	138 (75 %)	39 (78 %)	0.32
Moderate, n (%)	2 (1 %)	2 (1 %)	0 (0 %)	0.89
Aortic stenosis, n (%)	63 (20 %)	45 (25 %)	19 (38 %)	
Mild, n (%)	27 (8 %)	7 (4 %)	1 (2 %)	0.016
Moderate, n (%)	24 (7 %)	15 (8 %)	9 (18 %)	0.10
Severe, n (%)	12 (4 %)	11 (6 %)	9 (18 %)	0.002
Aortic regurgitation, n (%)	118 (37 %)	72 (39 %)	25 (50 %)	
Mild, n (%)	113 (35.4)	69 (37.9)	24 (48.0)	0.15
Moderate, n (%)	5 (1.6)	3 (1.6)	1 (2.0)	0.85

^a^Jonckheere-Terpstra Test

No significant differences in baseline characteristics were noted among subjects without any VHD and those with mild VHD. By contrast, higher rates of decompensated heart failure, stroke and chronic atrial fibrillation were noted in subjects with moderate-to-severe VHD as compared to those with no or mild VHD. The prevalence of heart failure related symptoms, such as dyspnea and peripheral edema were also higher among subjects with moderate-to-severe VHD than among those without (Table 2). Patients with moderate-to-severe VHD exhibited higher CIRS and GDS-15 scores, and lower ADL scores than those without. As shown in Table 3, patients with moderate-to-severe VHD exhibited larger LAs, LV masses, E/e’ ratios and E/A ratios, as well as shorter deceleration (DT) and isovolumic relaxation times than those without.

**Table 2:** Clinical characteristics of the study population

	No VHD (*n* = 92)	Mild VHD (*n* = 367)	Moderate-to-severe VHD (*n* = 97)	*p* value
Socio-demographic characteristics				
Male, n (%)	38 (41 %)	135 (37 %)	33 (34 %)	<0.001
Age, (years)	84.1 ± 2.9	84.6 ± 3.6	86.3 ± 4.3	0.58
Coronary risk factors and multimorbidity				
Smoking (current or ex), n (%)	31 (34 %)	122 (33 %)	22 (23 %)	0.11
Hypertension, n (%)	60 (66 %)	258 (70 %)	72 (74 %)	0.46
Dyslipidemia, n (%)	40 (45 %)	165 (46 %)	37 (39 %)	0.46
Type 2 diabetes, n (%)	21 (23 %)	62 (17 %)	19 (20 %)	0.38
CIRS, median (IQR)	4 (3–5)	3 (3–5)	4 (3–6)	0.008
Prior history				
Myocardial infarction, n (%)	17 (19 %)	35 (10 %)	8 (8 %)	0.029
TIA or stroke, n (%)	15 (16 %)	57 (16 %)	29 (30 %)	0.025
COPD, n (%)	13 (12 %)	40 (11 %)	10 (10 %)	0.79
Peripheral arterial disease, n (%)	10 (11 %)	29 (8 %)	11 (11 %)	0.45
CABG, n (%)	8 (9 %)	20 (5 %)	8 (8 %)	0.39
PCI, n (%)	12 (13 %)	30 (8 %)	5 (5 %)	0.13
Valvular surgery, n (%)	2 (2 %)	15 (4 %)	2 (2 %)	0.48
Heart failure, n (%)	8 (9 %)	36 (10 %)	16 (16 %)	0.14
Chronic atrial fibrillation, n (%)	4 (4 %)	34 (9 %)	19 (20 %)	0.002
Pacemaker, n (%)	5 (6 %)	21 (6 %)	5 (5 %)	0.97
Symptoms				
Angina pectoris, n (%)	11 (12 %)	58 (16 %)	21 (22 %)	0.20
Dyspnea ≥ 3^a^, n (%)	22 (24 %)	101 (28 %)	42 (43 %)	0.005
Peripheral edema, n (%)	31 (34 %)	115 (31 %)	42 (43 %)	0.090
Fatigue, n (%)	17 (19 %)	75 (21 %)	22 (23 %)	0.80
Cardiac medications				
Diuretics, n (%)	45 (49 %)	162 (44 %)	54 (56 %)	0.12
Potassium-sparing agents, n (%)	17 (18 %)	53 (14 %)	17 (18 %)	0.54
ACE inhibitors, n (%)	20 (22 %)	123 (28 %)	29 (30 %)	0.39
ARBs, n (%)	18 (20 %)	51 (14 %)	17 (18 %)	0.34
ß-blockers, n (%)	28 (30 %)	161 (44 %)	46 (47 %)	0.035
Digitalis, n (%)	0 (0 %)	11 (3 %)	11 (11 %)	<0.001
Physical activity and cognitive functioning				
ADL, median (IQR)	25 (21–27)	25 (21–28)	23 (17–27)	<0.001
MMSE, median (IQR)	28 (26–29)	28 (26–29)	28 (25–29)	0.36
GDS-15, median (IQR)	2 (1–4)	2 (1–4)	3 (2–5)	0.033

^a^according to the Medical Research Council dyspnea scale. *ACE* angiotensin converting enzyme, *ARB* angiotensin receptor blocker, *CABG* coronary artery bypass graft surgery, *CIRS* cumulative illness rating scale, *COPD* chronic obstructive pulmonary disease, *IQR* interquartile range, *PCI* percutaneous coronary intervention, *TIA* transient ischemic attack

**Table 3:** Echocardiographic characteristics of the study population

	No VHD (*n* = 92)	Mild VHD (*n* = 367)	Moderate-to-severe VHD (*n* = 97)	*p* value
LV morphology and function				
LVEF (%)	55 ± 3	55 ± 5	55 ± 3	0.60
LVEF < 50 %, n (%)	4 (4 %)	26 (7 %)	2 (2 %)	0.14
LV mass (g/m^2^)	86 ± 21	92 ± 23	98 ± 27	0.002
LVH, n (%)	10 (11 %)	57 (16 %)	18 (19 %)	0.33
Indexed LAV (mL/m^2^)	30 ± 9	32 ± 10	39 ± 12	<0.001
Indexed LAV > 34 mL/m^2^, n (%)	23 (25 %)	145 (40 %)	62 (65 %)	<0.001
Stroke volume (mL/beat)	68 ± 19	70 ± 16	68 ± 14	0.48
RWMA, n (%)	13 (14 %)	43 (12 %)	7 (7 %)	0.30
LV diastolic filling				
E/A ratio	0.87 ± 0.44	0.92 ± 0.48	1.07 ± 0.58	0.027
E/A ratio ≥ 1.5, n (%)	6 (7 %)	31 (9 %)	14 (19 %)	0.013
E/e’ ratio	10.7 ± 3.1	11.6 ± 4.0	13.5 ± 4.2	<0.001
E/e’ ratio ≥ 13, n (%)	23 (25 %)	102 (28 %)	41 (53 %)	<0.001
Deceleration time (ms)	178 ± 41	178 ± 44	174 ± 51	0.74
Deceleration time < 160 ms, n (%)	29 (32 %)	115 (32 %)	35 (48 %)	0.028
Vp (cm/s)	58 ± 17	56 ± 16	52 ± 22	0.12
Vp < 45 cm/s, n (%)	23 (25 %)	114 (32 %)	35 (48 %)	0.006

*A* atrial transmitral peak velocity, *E* early transmitral peak velocity, *e’* early peak mitral annular velocity, *LAV* left atrial volume, *LVEF* left ventricular ejection fraction, *LVH* left ventricular hypertrophy (LV mass index >109 g/m^2^ in women and >132 g/m^2^ in men.), *RWMA* regional wall motion abnormalities, *Vp* velocity of inflow propagation

### Clinical outcomes

**Fig. 1:**
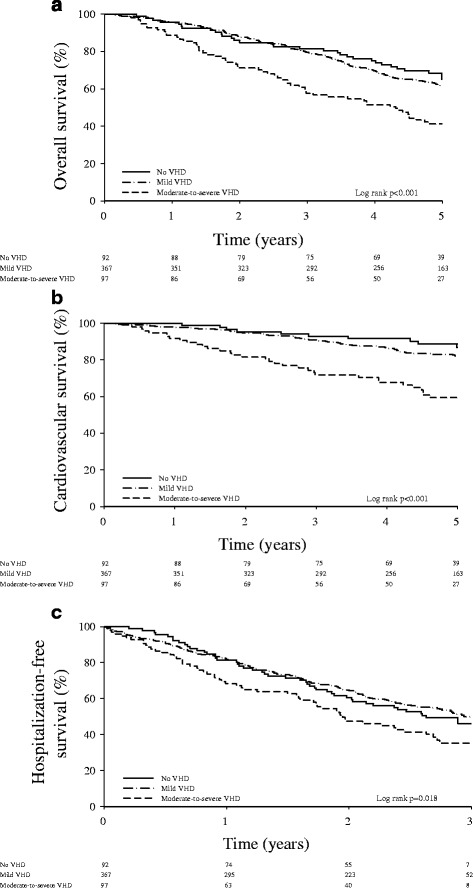
Kaplan Meier survival curves for overall survival (panel **a**), cardiovascular survival (panel **b**) and freedom from a first unplanned hospitalization (panel **c**) in patients with no (solid lines), mild (dotted-dashed lines) and moderate-to-severe (dashed lines), mild and without (solid lines) VHD. Risk of mortality and hospitalization based on the presence of valvular heart disease

Follow-up was 100 % complete. After an average follow-up time of 5.1 ± 0.3 years, there were 236 deaths (42 %), of which 102 (43 %) were of cardiovascular origin. Among the 97 subjects with moderate-to-severe VHD, 57 (59 %) died, of which 33 (58 %) died of cardiovascular causes. Among the subjects with VHD and systolic dysfunction, 19 (59 %) died, of whom 8 (42 %) from cardiovascular causes. As shown in Fig. 1, 5-year overall and cardiovascular survival was significantly lower in subjects with moderate-to-severe VHD than in those with no or mild VHD.

After an average follow-up time of 3.0 ± 0.3 years, 278 participants (50 %) needed hospitalization. Hospitalization data were missing for only one participant. As shown in Fig. 1c, the freedom from a first unplanned hospitalization was better in patients with no or mild VHD than in those with moderate-to-severe VHD.

### Predictors of all-cause and cardiovascular mortality

As show in Table 4, the multivariable Cox’s regression analysis identified age, moderate-to-severe VHD, CIRS, severe dyspnea, systolic dysfunction and LV hypertrophy as independent predictors of all-cause mortality. A dose-response effect was found for LV mass index (adjusted HR: 1.01, 95 %-CI [1.00–1.01], *p* = 0.003) but not for LV ejection fraction. No relation was found with parameters of diastolic dysfunction.

**Table 4:** Uni- and multivariable predictors of outcome

	Univariable			Multivariable		
	HR	95 % CI	*p* value	HR	95 % CI	*p* value
All-cause mortality						
Age (year)	1.10	1.07–1.14	<0.001	1.09	1.05–1.12	<0.001
Dyspnea ≥3	2.29	1.77–2.97	<0.001	1.81	1.37–2.39	<0.001
CIRS (per point increase)	1.21	1.12–1.30	<0.001	1.11	1.03–1.21	0.007
Moderate-to-severe VHD	1.91	1.42–2.58	<0.001	1.42	1.04–1.95	0.029
Moderate-to-severe AS	1.96	1.42–2.70	<0.001			
LAVI ≥34 mL/m^2^	1.58	1.22–2.04	0.001			
LV hypertrophy	1.01	1.00–1.01	0.004	1.01	1.00–1.01	0.020
Moderate-to-severe MS	2.30	1.08–4.88	0.030			
LVEF ≤50 %	1.63	1.02–2.61	0.040	1.66	1.03–2.67	0.037
Dyslipidemia	0.78	0.60–1.01	0.063			
Cardiovascular mortality						
Age (year)	1.09	1.04–1.14	<0.001	1.06	1.01–1.11	0.014
CIRS (per point increase)	1.23	1.10–1.37	<0.001			
Dyspnea ≥3	3.06	2.07–4.53	<0.001	2.68	1.80–3.98	<0.001
Moderate-to-severe VHD	2.88	1.90–4.37	<0.001	2.13	1.38–3.29	0.001
Moderate-to-severe AS	3.06	1.99–4.72	<0.001			
Moderate-to-severe MS	4.65	2.03–10.63	<0.001			
LAVI ≥34 mL/m^2^	1.87	1.27–2.77	0.002			
LV hypertrophy	1.01	1.00–1.02	0.029	1.01	1.00–1.01	0.087
First unplanned Hospitalization						
Smoker (current or ex)	1.60	1.25–2.04	<0.001	1.69	1.32–2.16	<0.001
CIRS (per point increase)	1.20	1.13–1.29	<0.001	1.15	1.07–1.23	<0.001
Dyspnea ≥3	1.81	1.42–2.31	<0.001	1.50	1.16–1.94	0.002
Moderate-to-severe AS	1.71	1.25–2.33	0.001			
LAVI ≥34 mL/m^2^	1.37	1.08–1.74	0.009			
Moderate-to-severe VHD	1.51	1.13–2.03	0.006	1.43	1.06–1.94	0.021
Age (year)	1.03	1.00–1.06	0.041			

*AS* aortic stenosis, *CI* confidence interval, *CIRS* cumulative illness rating scale, *HR* hazard ratio, *LAVI* left atrial volume index, *LVEF* left ventricular ejection fraction, *MS* mitral stenosis, *VHD* valvular heart disease

Moderate-to-severe VHD was also identified as an independent predictor of cardiovascular mortality (adjusted HR: 2.13; 95 %-CI [1.38–3.29]), together with age, CIRS, LV hypertrophy and severe dyspnea (Table 4). A dose-response effect was found for both LV hypertrophy (adjusted HR: 1.01; 95 %-CI [1.00–1.02], *p* = 0.029). Diastolic parameters did not show any relationship with cardiovascular mortality.

### Predictors of first unplanned hospitalizations

Moderate-to-severe VHD, together with the smoking status, CIRS, and severe dyspnea, was found to be independently associated with the need for a first unplanned hospitalization.

The relationships between moderate-to-severe VHD and all-cause mortality, cardiovascular mortality and the need for a first unplanned hospitalization did not change when echocardiographic parameters were entered as dichotomous or continuous variables, nor when the CIRS was replaced by individual morbidities.

## Discussion

Studies investigating the prevalence of VHDs in elderly subjects are rather scarce and often based on in-hospital series [4, 20–22]. Studies describing the clinical impact and outcome consequences of VHDs in very old subjects are even fewer [22], making it difficult to appreciate their real burden in our ageing societies. The current study is the first to investigate the prevalence, clinical impact and outcome consequences of VHDs in a large population-based sample of subjects aged 80 and older. Our results can be summarized as follows:

- The prevalence of moderate-to-severe VHD in very old subjects is around 17 %, and is similar in men and women.
- There is a significant age-related increase in both the prevalence and the severity of aortic stenosis.
- Higher rates of heart failure, atrial fibrillation and stroke were found in subjects with moderate-to-severe VHD than in those without. Accordingly, physical activity and cognitive functioning was negatively affected in patients with moderate-to-severe VHD.
- The presence of moderate-to-severe VHD independently increases overall and cardiovascular mortality risks, as well as the need for unplanned hospitalizations.

### Prevalence of VHD in very old subjects

The BELFRAIL study is one of the largest prospective observational studies performed so far on the prevalence of VHD in very old subjects. The salient finding of this study is that the prevalence of moderate-to-severe VHD in subjects aged 80 and older is quite high, in the range of 16−18 %. Moderate-to-severe AS was by far the most frequently encountered VHD (14 % of very old subjects), followed by moderate-to-severe degenerative MS (1.7 %), moderate AR (1.6 %) and finally moderate MR (0.7 %). MS was more prevalent in women and an age-dependent increase in the prevalence and severity of AS was observed.

Few previous studies have investigated the prevalence of VHD in elderly subjects. Although these studies demonstrated an age-dependent increase in the prevalence of VHD in the ageing population, very little data are available concerning VHD prevalence among very old subjects, i.e. those aged 80 years and older. By using data from 3 large north-American population-based studies [23–25] as well as from the Olmsted county community study, Nkomo et al. reported a prevalence of moderate-to-severe VHD in Americans aged 75 years and older of 11.7 to 13.2 % [4]. Interestingly, most of their patients presented with moderate-to-severe MR (9.3 % in the population-based studies, 7.1 % in the Olmsted County community study) whereas fewer patients presented with moderate-to-severe AS (2.8 % in the population-based studies, 4.6 % in in the Olmsted County community study). Although the results of our study are roughly in line with those of Nkomo’s study, they nonetheless differ with regard to the relative prevalence of MR and AS, MR being more prevalent in these earlier studies than in ours. Our results also differ from those of the Leyden 85+ study, in which the prevalence of moderate-to-severe VHD among subjects aged 90 and older was found to be close to 50 %, the majority of the subjects presenting with moderate to severe MR, and only 6 % with moderate-to-severe AS [22]. The reasons for these differences are unclear but may be related to the methods used to assess of MR severity. Most previous studies, including that from Nkomo et al., used the semi-quantitative method described by Helmcke et al [26] to make this assessment. While this approach works reasonably well in patients with central jets, it is notoriously inaccurate in those with impinging wall jets due to mitral valve prolapse [27]. In the present study, we used an integrated approach that included both qualitative and quantitative parameters, in order to minimize the natural tendency of echocardiographic methods to overestimate the true severity of MR [28].

### Clinical burden of VHD in very old subjects

Another important objective of our study was to investigate the clinical and echocardiographic characteristics of very old subjects presenting with VHD. Our findings indicate that patients with moderate-to-severe VHD more often present with heart failure symptoms, such as dyspnea or peripheral edema; more frequently have a prior history of heart failure and stroke; and more frequently present with atrial fibrillation. This negatively affected their physical activity and cognitive functioning as measured by the ADL and GDS-15 scores. They were also more likely to exhibit increased LA volume and LV mass and to demonstrate echocardiographic features associated with altered LV diastolic properties and elevated filling pressures.

Our data also indicate that the VHD detected in our study were not simply benign imaging observations but had profound outcome consequences, as they were associated with a 40 % increase in adjusted overall mortality and a 2.1-fold excess in adjusted cardiovascular mortality. Patients with moderate-to-severe VHD were also hospitalized more frequently than patients without. Our observations confirm and extend those made by Nkomo et al. as they demonstrate that in elderly patients as well, moderate-to-severe VHDs are a major and independent determinant of outcome. Our results also concur with those of studies that specifically addressed the impact of aortic stenosis on the outcome of elderly patients [29, 30].

The excess risk associated with VHD stresses the importance of increasing the awareness of the medical community on the dismal outcome consequences of VHDs and on the need to detect them early. Early detection of VHD, before the onset of symptoms and at a time when patients are still sufficiently fit to be proposed surgical valve replacement is probably key to improving their prognosis. Nkomo et al. have indeed shown that in the community, VHDs are frequently underdiagnosed, owing probably to the need for echocardiography to be clinically indicated before diagnosis is made [4]. Whereas delaying diagnosis likely has little impact on outcome in younger individuals, it exposes elderly patients to be denied appropriate treatment because of their advanced age. The EuroHeart survey has indeed clearly demonstrated that old patients are frequently denied interventions despite unequivocal guideline-based indications, because of their advanced age [10, 11].

### Study limitations

This study has limitations that should be acknowledged. First, although many factors potentially associated with VHDs were considered, we cannot exclude the possibility that some yet unknown confounders contributed to our results. Second, comorbidities may have been underdiagnosed, as their identification relied only on the information gathered by the general practitioner.

## Conclusions

In this large representative population-based sample of subjects aged 80 and older a high prevalence of VHD was found. Patients with VHD more frequently presented with heart failure symptoms, atrial fibrillation and stroke than those without. They were also found to be at higher risk for all-cause and cardiovascular mortality as well as unplanned hospitalizations.
